# Integrative Analysis of N6-methyladenosine RNA modifications related genes and their Influences on Immunoreaction or fibrosis in myocardial infarction

**DOI:** 10.7150/ijms.86210

**Published:** 2024-01-01

**Authors:** Shiwei Zhu, Lan Bai, Yitong Pan, Junhao Yin, Deshuai Zhang, Chenchen Hou, Yongli Wang, Ruogu Li

**Affiliations:** 1 Department of Cardiology, Shanghai Chest Hospital, Shanghai Jiao Tong University School of Medicine, Shanghai 200030, China.; 2 Laboratory of Oral Microbiota and Systemic Diseases, Shanghai Ninth People's Hospital, College of Stomatology, Shanghai Jiao Tong University School of Medicine, Shanghai 200125, China.; 3 National Center for Stomatology; National Clinical Research Center for Oral Diseases; Shanghai Key Laboratory of Stomatology, Shanghai 200011, China.; 4 Department of Oral Surgery, Shanghai Ninth People's Hospital, Shanghai Jiao Tong University School of Medicine, Shanghai 200011, China.; 5 Department of Respiratory and Critical Care Medicine, Shanghai Ninth People's Hospital, Shanghai Jiao Tong University School of Medicine, Shanghai 200011, China.

**Keywords:** MI, m6A regulation, immune response, fibrosis, LRPPRC, WGCNA

## Abstract

Increasing studies have shown that N6-methyladenosine (m6A) modification plays an important role in cardiovascular diseases. In this study, we systematically investigated the regulatory mode of m6A genes in myocardial infarction (MI) by combining bioinformatics analysis of clinical samples with animal experiments. We utilized gene expression data of clinical samples from public databases to examine the expression of m6A genes in heart tissues and found a large difference between the healthy control group and MI group. Subsequently, we established an MI diagnosis model based on the differentially expressed m6A genes using the random forest method. Next, unsupervised clustering method was used to classify all MI samples into two clusters, and the differences in immune infiltration and gene expression between different clusters were compared. We found LRPPRC to be the predominant gene in m6A clustering, and it was negatively correlated with immunoreaction. Through GO enrichment analysis, we found that most differentially expressed genes between the two clusters were profibrotic. By means of WGCNA, we inferred that GJA4 might be a core molecule in the m6A regulatory network of MI. This study demonstrates that m6A regulators probably affects the immune-inflammatory response and fibrosis to regulate the process of MI, which provides a potential therapeutic target.

## 1. Introduction

Myocardial infarction (MI) is the necrosis of the myocardium caused by coronary artery obstruction and continuous ischemia and hypoxia^1^. It can result in heart failure, thrombosis and malignant arrhythmia, which threatens the life of patients 2. With the increasing incidence of coronary heart disease, the threat caused by MI to human health has become progressively serious. Currently, the main treatment strategy for MI is to improve heart blood supply, including surgical operation and drug treatment^3^. It has recently been reported that the primary goal of surgical revascularization is to prevent further injury by protecting the remaining viable myocardium from subsequent acute coronary events. However, a myriad of signaling pathways play a role from the onset of MI to heart failure, including regulating myocardial apoptosis after myocardial infarction, immune-inflammatory responses, myocardial fibrosis, and angiogenesis, and these pathways provide a number of potential therapeutic targets for MI^4^.

m6A modification is the most common RNA epigenetic modification in eukaryotic cells, and it is a reversible process that regulates RNA transcription, splicing, degradation, and translation without altering the base sequence^5^. The regulators of m6A modification can be divided into three categories, namely, methyltransferases, demethylases, and methylation readers^6^. Methyltransferases, including METTL3, METTL14, and WTAP, assemble into protein complexes and catalyze adenylate m6A modification on RNA. FTO and ALKBH5 are the only two components of demethylases, which catalyze the demethylation reaction of m6A-modified bases. Methylation readers, including YTHDC1, YTHDC2, and HNRNPC, are RNA-binding proteins that specifically bind to the region of m6A modification, change the secondary structure of RNA, and affect the interaction between RNA and protein.

m6A modification has been confirmed to play an important role in cardiac physiology and the occurrence and development of a variety of cardiovascular diseases^7^, ^8^. There are large differences in m6A methylation of RNA between normal and failing hearts^9^. In general, methyltransferases tended to exacerbate heart function while demethylases have the opposite physiological effect. For example, METTL3, which is a widely studied m6A methyltransferases, may accelerate the apoptosis in hypoxia-treated cardiomyocytes and enhance autophagy^10^, weaken the repair capacity of infarcted hearts^11^ and exacerbates cardiac fibrosis by promoting fibroblast activation^12^. In addition, m6A modification mainly mediated by METTL3 enhances the responsiveness of cardiomyocytes to hypertrophy-promoting stimuli, thereby promoting cardiac hypertrophy^13^. Another methyltransferase, METTL14, enhances FOXO1 mRNA methylation to upregulate its expression, which induces an inflammatory response in vascular endothelial cells and exacerbates atherosclerosis^14^. In contrary, the expression of the m6A demethylase, FTO, is reduced in failing mammalian hearts and hypoxic cardiomyocytes, thereby increasing m6A modification of RNA and reducing the contractile function of cardiomyocytes^15^. This protective effect of FTO on cardiac function is exerted partly through the regulation of glucose uptake and glycolysis^16^. FTO an also induces demethylation of Yap1 mRNA to increase its stability, which attenuates ischemia-induced cardiomyocyte apoptosis^17^. Another demethylase, ALKBH5, was also proved to play a protective role in MI^10^. Besides, numerous studies have demonstrated that m6A modification is involved in different stages of organ scar formation, and the underlying mechanism depends on the synergistic effects of writers, erasers, and readers upon gene expression and ncRNA maturation. siRNAs or drugs targeting these proteins have shown significant antifibrotic activity in vitro and in vivo^18^.

In the present study, we systematically investigated the expression of m6A genes in MI to determine whether m6A modification functions in this disease. We analyzed the differentially expressed genes (DEGs) between normal heart tissue and MI heart tissue, and we screened the most important genes in the disease using the random forest method. We then used a nomogram to predict the disease incidence. Based on the expression of m6A genes in the disease group, we used unsupervised consensus clustering to cluster MI samples and analyzed immune cell infiltration between different subtypes. In addition, we identified the DEGs between different subtypes, and we analyzed the enriched pathways of these genes. Thus, the present study provides a comprehensive analysis of the role of m6A modification in MI and suggests potential research targets for the treatment of MI.

## 2. Materials and Methods

### 2.1. Raw data collection and preprocessing

We first searched the GEO database and downloaded two datasets, namely, GSE5406 and GSE57338, both of which contain gene expression of human heart tissues. GSE5406, which used the Affymetrix Human Genome U133A Array (GPL96) platform, contains 16 healthy control samples, 108 samples with ischemic cardiomyopathy, and 86 samples with idiopathic cardiomyopathy. GSE57338, which used the GPL11532 ([HuGene-1_1-st] Affymetrix Human Gene 1.1 ST Array [transcript (gene)] version] platform), contains 136 healthy control samples, 95 samples with ischemic cardiomyopathy, and 82 samples with idiopathic cardiomyopathy. We first removed idiopathic cardiomyopathy samples from these two datasets because idiopathic cardiomyopathy was not part of the present study. After merging the data, all probes were labeled with corresponding gene names, and those lacking annotations were eliminated. We used the limma package to perform quantile normalization of the expression data. Subsequently, we utilized the SVA package to remove batch effects from both datasets, obtaining a final total gene expression matrix containing 152 healthy control samples and 203 MI samples. The analysis and mapping of this gene expression matrix was mainly conducted on the Sangerbox website (http://www.sangerbox.com/tool)^19^.

### 2.2. Screening and extraction of MI-related m6A genes

We summarized and sorted most of the m6A genes and classified them into writers, readers, and erasers according to previous studies^20^, ^21^. We then extracted the expression data of m6A genes in all samples using the limma package in R software, and we screened the m6A genes that were differentially expressed between the normal control group and the MI group based on the following criteria: adjusted p value<0.05 and |log2 (fold change)| > 1. Pearson correlation analysis was used to determine the correlation between different m6A genes.

### 2.3. Construction and validation of the diagnostic model

The random forest (RF) method and support vector machine (SVM) method were used to identify the most suitable algorithm for establishing the diagnostic model of MI. RF is an ensemble learning method that integrates many decision trees into a forest to predict outcomes. In the RF method, the importance of variables is indicated by the lower Gini coefficient. SVM is a transformable machine learning algorithm that transforms previously indivisible data into linearly separable data through a kernel function. The importance of the supporting SVM variable is determined by the discriminant function coefficient value, w2. These two methods were implemented using the randomForest and kernlab packages in R software. We used DALEX packages to compare the residuals between the two models to determine the best model. After dimension reduction and feature selection, the selected m6A regulators were used to construct a prediction model by logistic regression analysis. We then used calibration curves, DCA curves, and clinical impact curves to investigate the accuracy of the prediction model.

### 2.4. Unsupervised cluster analysis of m6A modification patterns in MI

We used an unsupervised approach to classify MI samples into distinct m6A modification patterns based on the expression of 20 m6A genes. We then utilized the ConsensusClusterPlus package to construct the cumulative distribution function curves corresponding to k=2-9 and delta area score, from which the most appropriate number of clusters was confirmed. Subsequently, principal component analysis (PCA) was used to verify the clustering effect of these two m6A modification subgroups. Additionally, differentially expressed genes (DEGs) in MI samples from different m6A clusters were evaluated to identify genes mediated by m6A regulators (p<0.05).

### 2.5. Establishment of the MI model

We used a coronary artery ligation method to establish an MI model^22^. Briefly, 8-week-old adult C57BL/6 mice were selected and anesthetized with 2-3% isoflurane by inhalation in the induction chamber. The mice were then moved from the induction chamber to the surgical plate, secured with tape, and continuously anesthetized with 2% isoflurane through a coaxial breathing device. The chest skin was depilated and sterilized with medical alcohol. A small skin incision (1.2 cm long) was made over the left chest with scissors, and the pectoralis major and pectoralis minor muscles were separated to expose the fourth intercostal space. A small hole was further made in the fourth intercostal space with mosquito forceps to penetrate the intercostal muscle, pleura, and pericardium, and the heart was subsequently extruded. The descending branch of the main left ventricular coronary artery was located and ligated with a 7-0 suture. After ligation, the heart was pushed back into the chest cavity, and the gap in the chest wall was closed. The chest cavity was then gently squeezed to expel gas in case of a pneumothorax, and the skin incision was purse-string sutured.

### 2.6. Cardiac RNA extraction, reverse transcription, qPCR, and RNA-Seq

Total RNA was extracted from heart tissue with TRIzol reagent (Life Technologies/Thermo Fisher Scientific). One portion of the RNA sample was sent to Biomarker Company for RNA-seq analysis, and the remaining RNA was used for subsequent reverse transcription and qPCR. RNA samples (500 ng) were converted into cDNA using a reverse transcription kit (Takara, Shiga, Japan). SYBR Green Mix (Life Technologies/Thermo Fisher Scientific) was used for quantitative reverse transcription PCR using an ABI7900HI system (Applied Biosystems/Thermo Fisher Scientific). The expression of target genes was normalized to the GAPDH reference gene.

### 2.7. Immune cell infiltration and fibrosis assessment

We used the GSVA package in R software to perform single-sample gene set enrichment analysis (ssGSEA) on human and mouse gene expression profiles to evaluate the infiltration levels of various immune cells, and we then used a t test to detect differences in ssGSEA scores between different m6A clusters in humans or mice to infer differences in immune cell infiltration.

For fibrosis assessment, the hearts of mice 4 weeks after MI model establishment were harvested and cut perpendicular to the longitudinal axis of the heart at the ligation point. The bottom of the heart was used for RNA extraction, and the apex half was fixed with 4% paraformaldehyde and sent to Ruchuang Biological Co. for paraffin embedding. The samples were then routinely sectioned and deparaffinized for Sirius Red staining with a Sirius Red staining kit according to the manufacturer's instructions, and the finished products were sealed with neutral gum. The areas of fibrosis were visualized with Sirius red staining.

### 2.8. GO enrichment analysis and WGCNA

To explore the biological function of DEGs between different m6A clusters, we performed Gene Ontology (GO) analysis. We used the org.Hs.eg.db package in R software to annotate the genes, and we used clusterProfiler in R for the enrichment analysis (p<0.05).

To identify hub genes, we applied weighted gene coexpression network analysis (WGCNA), an analytical method designed to identify coexpressed gene modules and explore associations between gene networks and phenotypes of interest. We used the WGCNA package in R software for analysis based on the gene expression profiles of MI samples. The outliers of genes and samples were removed by the goodSamplesGenes function of the WGCNA package. Pearson correlation analysis was used to analyze the correlation between different modules and subgroups. We further calculated the correlation between m6A modification patterns and gene expression to obtain gene significance (GS), and we used the relevant signature genes and gene expression profiles of modules to obtain module members (MMs).

### 2.9. Statistical analysis

Statistical analyses were performed using R v4.1.1 and Bioconductor software. All statistical tests were two-tailed, and a statistical threshold of α=0.05 was used throughout the research. Significant differences were annotated as follows: *p < 0.05; **p < 0.01; ***p < 0.001, or ****p < 0.0001.

## 3. Results

### 3.1. Expression of m6A regulators in myocardial infarction

**Figure 1:**
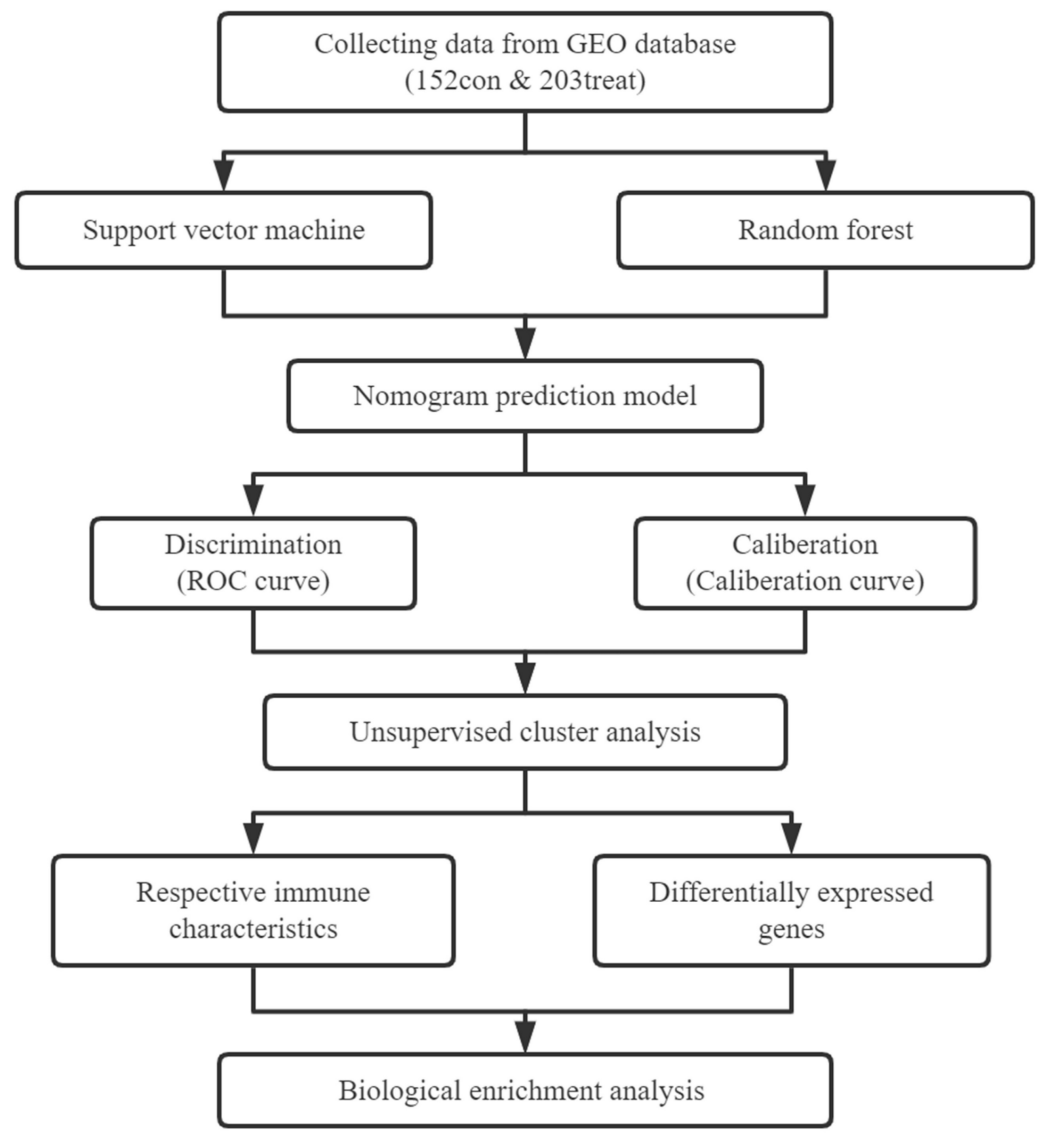
Flow chart of our research.

**Figure 2:**
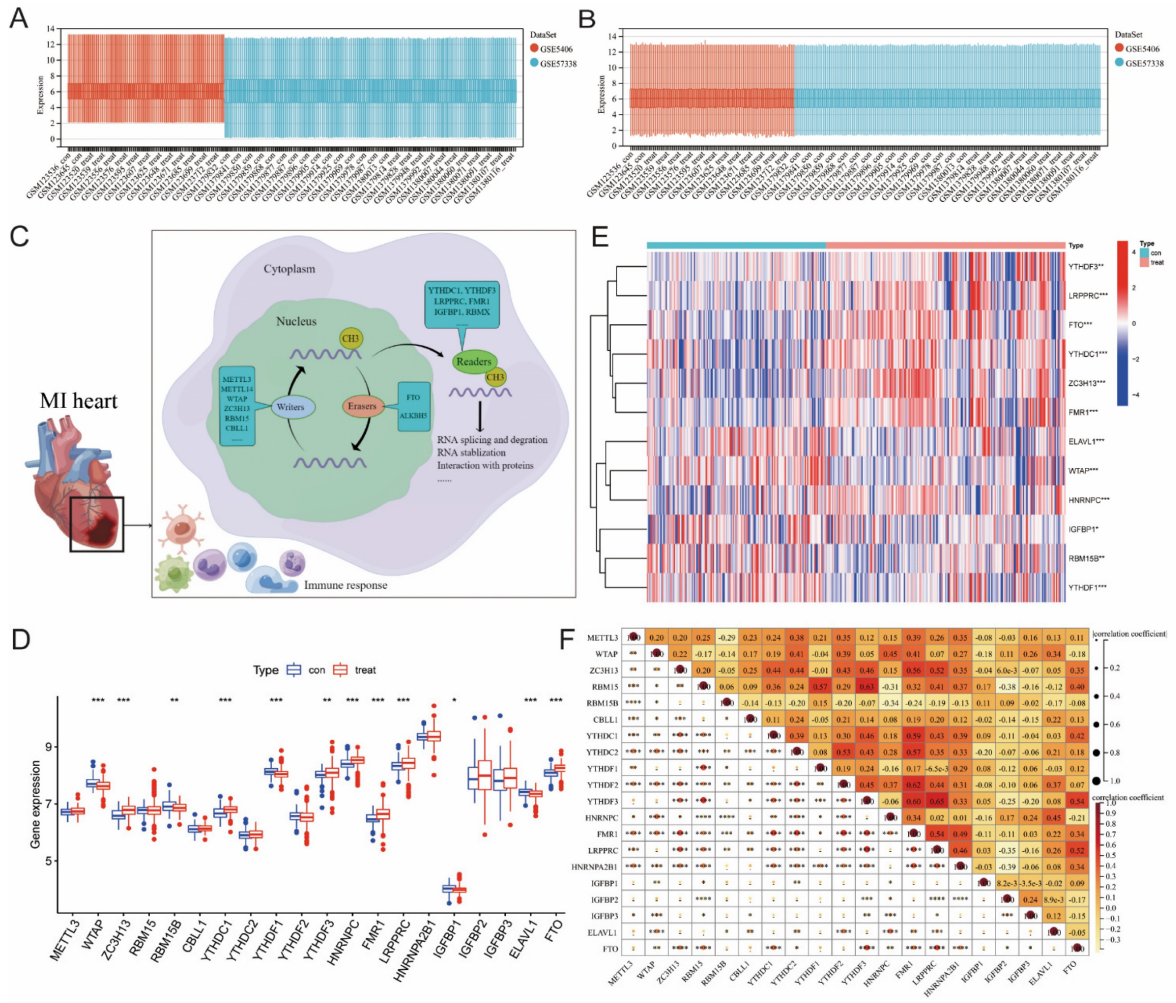
** Extraction and identification of differentially expressed m6A genes.** B) Gene expression distributions for both datasets before removing batch effects (A) and after removing batch effects (B). (C) Schematic representation of biological functions of m6A regulators in hearts of MI patients. (D) The box plot showed the difference in 20 m6A genes between the MI group and the healthy control group. (E) The heatmap of 12 differentially expressed m6A genes between the MI group and the healthy control group. (F) Correlation analysis of the expression of these 20 m6A genes. (The significance of difference is marked as follows: *p<0.05, **p<0.01, ***p<0.001).

Figure 1 illustrates our analysis procedure. We first used the inSilicoMerging package in R to combine the GSE5406 and GSE57338 datasets, and we used the method previously published by Johnson WE et al. to remove batch effects from the combined datasets to obtain the gene expression matrix without batch effects^23^. The data distribution before and after removing the batch effect indicated that the data distribution of the two datasets tended to be consistent (Figure 2A-B). The complete gene expression matrix is displayed in Additional file 2. We next sorted the 26 m6A regulators and classified them according to m6A writers, readers and erasers (Additional file 1, Table S1), and we used a schematic diagram to illustrate the dynamic process of m6A modification in MI heart tissues (Figure 2C). First, we extracted the expression of m6A regulators from all the samples of the dataset, resulting in a total of 20 m6A regulators (Figure 2D). Among these m6A regulators, the expression level of HNRNPA2B1 was significantly higher than that of the other 19 genes. There were 12 differentially expressed genes (DEGs) between the healthy control group and the MI group, namely, WTAP, ZC3H13, RBM15B, YTHDC1, YTHDF1, YTHDF3, HNRNPC, FMR1, LRPPRC, IGFBP1, ELAVL1, and FTO. Among these DEGs, the expression levels of ZC3H13, YTHDC1, YTHDF3, HNRNPC, FMR1, LRPPRC, and FTO were upregulated in the MI, while the expression levels of WTAP, RBM15B, YTHDF1, IGFBP1, and ELAVL1 were downregulated in the MI. We generated a heatmap to visualize the overall expression of these 12 DEGs in the two groups (Figure 2E), and we then performed a correlation analysis of the expression of these 20 m6A regulators to determine the internal association among them (Figure 2F). Positive correlations were found among YTHDF3, FMR1, LRPPRC, and most other m6A regulators. In addition, the correlation coefficient between YTHDF3 and LRPPRC was the highest (r=0.65).

### 3.2. Construction of a diagnostic model of MI based on m6A

**Figure 3:**
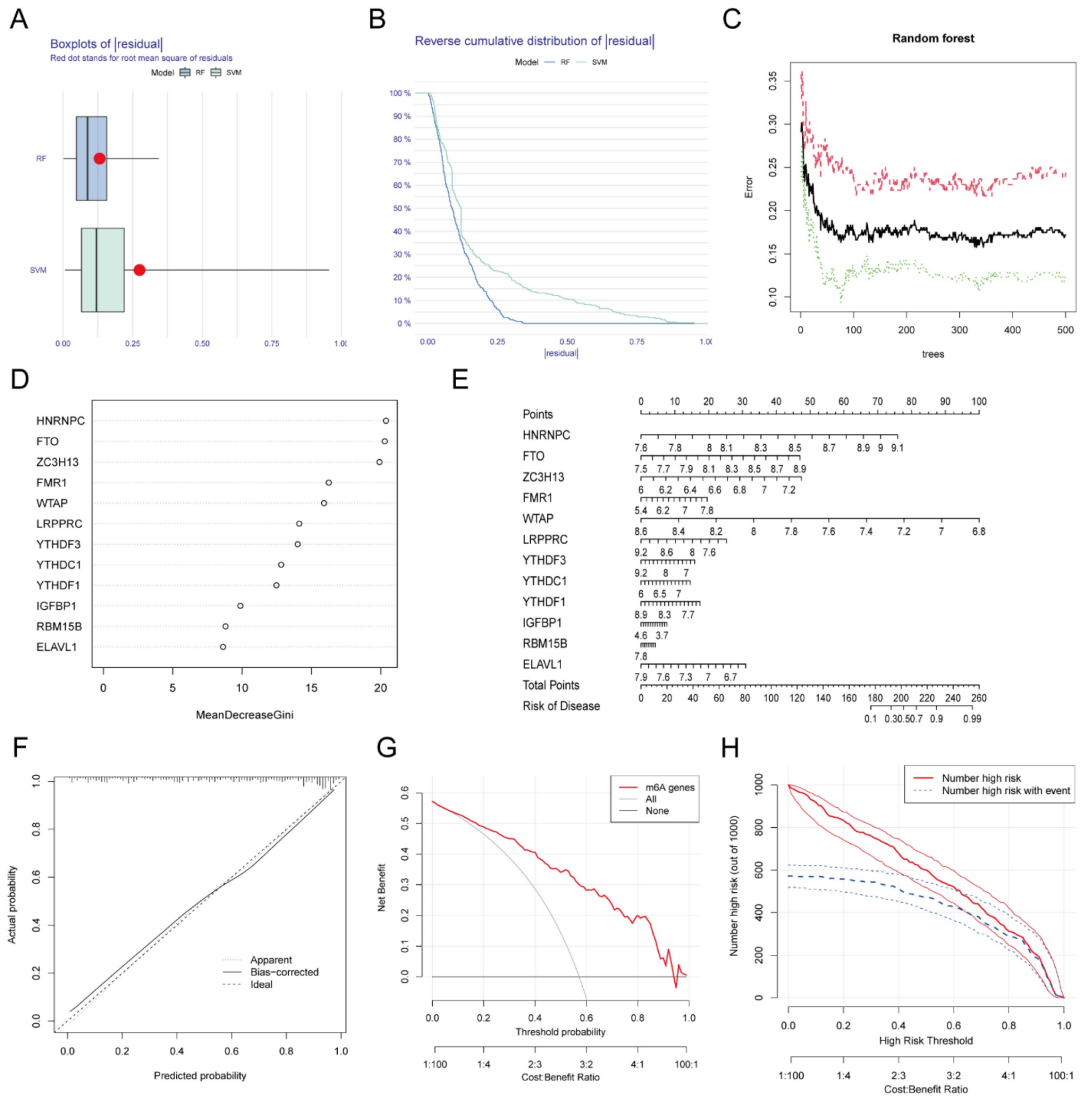
** Establishment and validation of the diagnostic model for MI.** The residual boxplot based on random forest (RF) and support vector machine (SVM) algorithms. (B) The plot of sample cumulative residuals based on RF and SVM. A small number of outliers can produce a lot of residuals (deviations from the true value), and for a large number of samples, the higher position of the line indicates the larger residuals. (C) Screening biomarkers based on RF. As the number of classification trees increases, the classification error decreases and the model tends to be stable. (D) Gini index of each predictor in the RF model, which indicates the importance of predictors. (E) The nomogram used for predicting the incidence of MI based on the expression of the 12 predictors. (F) Calibration curve used for testing the predictive ability of the nomogram model. (G) Decision curve analysis (DCA) curve to evaluate the accuracy of the nomogram model. (H) Clinical impact curve of the nomogram model.

We used RF and SVA algorithms to construct two models based on these 12 DEGs. According to our calculation results, the median residual of the RF algorithm was lower than that of the SVM algorithm (Figure 3A-B), which indicated that the MI diagnosis model established by the RF algorithm was more accurate. Figure 3C shows the relationship between the number of RF iterations and the classification error, which decreased and was more stable when the number of iterations reached approximately 350. We next calculated the Gini coefficients of these 12 DEGs, which were all greater than 2. Thus, we designated these 12 DEGs as predictors of MI (Figure 3D). We generated a nomogram based on these predictors using the rms package (Figure 3E) as a predictive model for the incidence of MI. To investigate the accuracy of the prediction model, we plotted the calibration curve, DCA curve, and clinical impact curve (Figure 3F-H), which revealed that the nomogram model had high value in the prediction of MI. In addition, we verified the consistency of these m6A gene changes in mice with those in humans. We established MI models in several C57BL/6 mice, sacrificed them one week later, and isolated RNA from their heart tissue. RNA was also extracted from normal mice simultaneously. The expression of the top eight most important m6A genes (i.e., Hnrnpc, Fto, Zc3h13, Fmr1, Wtap, Lrpprc, Ythdf3, and Ythdc1) in these samples were examined, and a heatmap was generated to visualize the results (Additional file 1, Figure S1). The changes in these genes in mice were consistent with those in humans at a first approximation, which further confirmed the feasibility of these genes as key genes for the diagnosis of MI.

### 3.3. m6A clusters of MI

**Figure 4:**
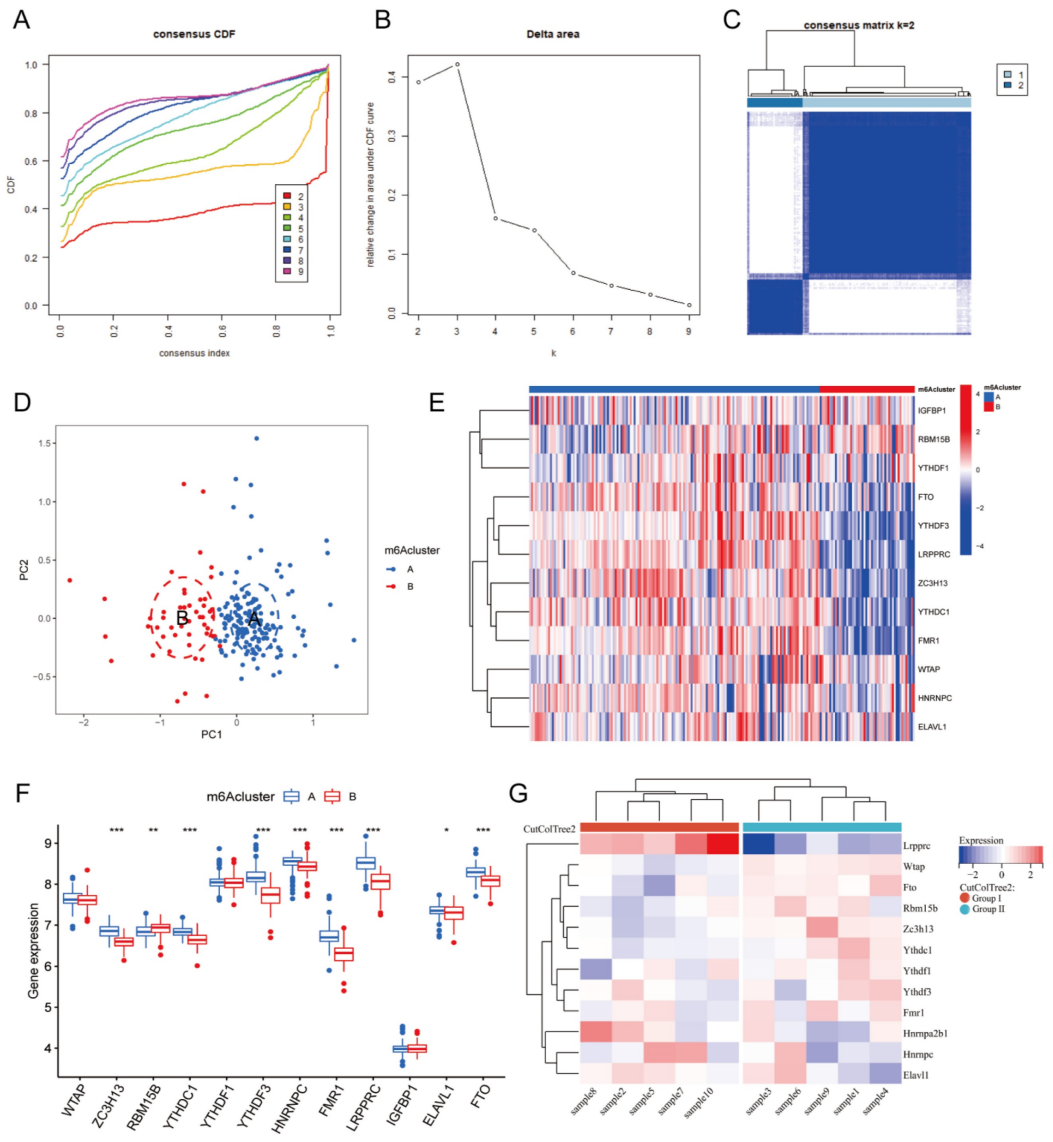
** m6A clusters of human and mouse MI samples.** Cumulative distribution function (CDF) curve of each consensus matrix from k=2 to 9. (B) Relative changes in the area under CDF curve based on different k values. (C) Consensus clustering of the 203 MI samples for k=2. (D) Principal component analysis (PCA) of the transcriptome profiles of the two m6A clusters, which revealed significant differences between these two m6A clusters. (E) Heatmap of 12 m6A regulators in different m6A clusters. (F) The expression of 12 m6A regulators in the two m6A clusters. (G) Heatmap of the 12 m6A regulators in mice with MI model. (The significance of difference is marked as follows: *p<0.05, **p<0.01, ***p<0.001).

Based on the expression of the 20 m6A genes in the samples of the MI group, we used an unsupervised clustering method to divide the dataset into different categories, and we then obtained the m6A clusters of the MI samples (Figure 4A-C, Additional file 3). After comparison, we found that k=2 was the most suitable clustering method. Thus, we divided all MI samples into two clusters as follows: 153 samples in Cluster A, and 50 samples in Cluster B. Next, we used PCA to visually demonstrate the similarity and correlation between Clusters A and B (Figure 4D). We then analyzed and compared the expression of each m6A regulator in these two subtypes and drew box plots and heatmaps (Figure 4E-F). In Cluster B, the expression of ZC3H13, YTHDC1, YTHDF3, HNRNPC, FMR1, LRPPRC, ELAVL1, and FTO was significantly decreased, while the expression of RBM15B was significantly increased. Moreover, YTHDF3, FMR1, and LRPPRC were the most differentially expressed m6A genes between the two clusters. Most of these differentially expressed m6A genes were m6A readers, which interact with each other to form a tightly linked regulatory network that controls a variety of molecular biological processes, such as mRNA degradation, mRNA stability, and translation enhancement^6^. The m6A cluster results further confirmed that MI is closely related to m6A modification.

To further support the explanation and confirmation of these results, we established MI models in 10 C57BL/6 mice. At one week after MI, the mice were sacrificed, and their hearts were harvested for RNA-seq analysis. An expression matrix of all genes in the 10 mice was obtained (Additional file 4). Subsequently, we extracted the expression of the 12 differentially expressed m6A genes and generated a heatmap (Figure 4G). According to the gene expression level, these 10 samples were clustered automatically into two groups, namely, Group I and Group II. The expression of Lrpprc in Group I was significantly higher than that in Group II, and this difference was more significant than that of the other m6A regulators. In the m6A cluster of clinical samples, the expression level of LRPPRC in Cluster A was significantly higher than that in Cluster B. Subsequent experiments will be performed to further elucidate the association between the clinical grouping and mouse sample typing.

### 3.4. Immunological differences between different m6A clusters of MI samples

**Figure 5:**
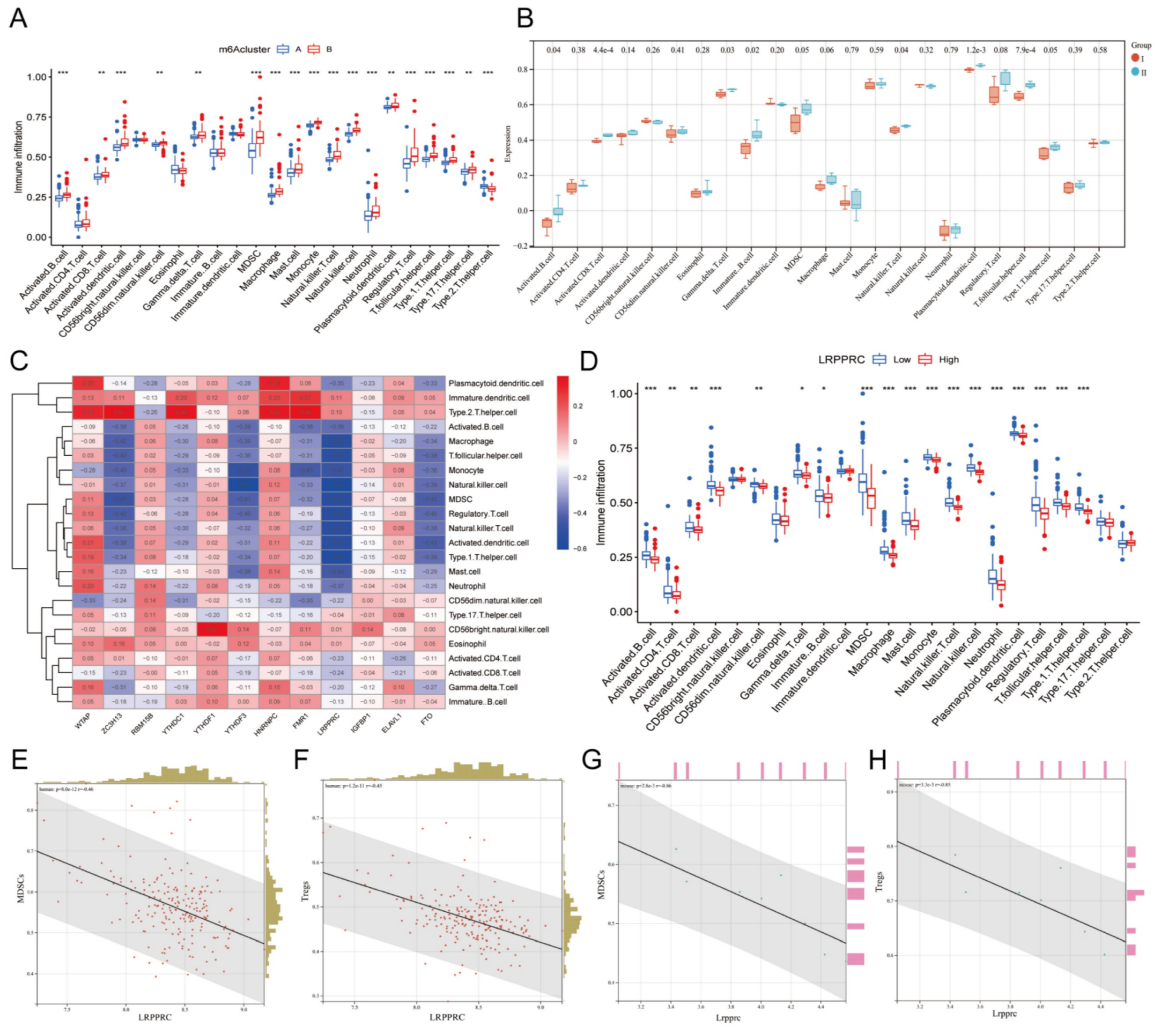
** Immunological characteristics of different m6A clusters.** Differences in immune cell infiltration between two different m6A clusters in human MI samples. (B) Differences in immune cell infiltration between two different m6A groups in mouse MI samples. (C) Heatmap of the correlation between these 12 m6A genes and each type of immune cells. (D) Differences in infiltration of immune cells between LRPPRC high expression group and LRPPRC low expression group. (E) Correlation analysis between LRPPRC expression and MDSC infiltration in human MI samples. (F) Correlation analysis between LRPPRC expression and Treg infiltration in human MI samples. (G) Correlation analysis between Lrpprc expression and MDSC infiltration in mouse samples. (H) Correlation analysis between Lrpprc expression and Treg infiltration in mouse samples.

The immune response plays an important role in the development of MI from the onset to the late stage^24^. To further investigate the relationship between m6A regulators and the immune microenvironment, we conducted ssGSEA of all samples based on the gene expression characteristics of various immune cells (Additional file 5), and the ssGSEA score was used to represent the infiltration of immune cells (Additional file 6). We then compared the infiltration of immune cells between Clusters A and B in human samples (Figure 5A) or Groups I and II in mouse samples (Figure 5B) and found that most of the immune cells demonstrated a significant distinction between the two clusters in human samples, especially MDSCs and Tregs. In general, the infiltration level of proinflammatory immune cells in Cluster B was higher than that in Cluster A, indicating that Cluster B may suffer a more severe inflammatory response. However, due to the small number of mouse samples, the infiltration of many types of immune cells did not show significant differences in mice. Nevertheless, we still found that the variation trend of most immune cells in mouse samples was consistent with that in human samples when considering Group I to be equivalent to Cluster A and group II to be equivalent Cluster B, indicating that the most significant distinctions were identified for MDSCs and Tregs.

To investigate which m6A regulator has the greatest impact on immune cell infiltration, we generated a heatmap of the correlation between the differentially expressed m6A genes and immune cell infiltration in human MI samples (Figure 5C). To our surprise, LRPPRC, the m6A reader that was most distinct in m6A clusters, was negatively correlated with the majority of infiltrating immune cells. Thus, we further compared the differences in immune cell infiltration between the groups with high and low expression of LRPPRC, which demonstrated that the immune cell infiltration in the group with high LRPPRC was significantly lower with the most distinct variation in MDSCs and Tregs (Figure 5D). In this regard, we analyzed the correlation between the expression of this m6A reader and MDSC or Treg infiltration in human MI samples and MI-exerted mouse heart samples (Figure 5E-H). In both human and mouse samples, Lrpprc (or LRPPRC) expression showed an inverse correlation with MDSC and Treg infiltration. In addition, YTHDF3 expression was also negatively correlated with the majority of immune cells, while WTAP and HNRNPC expression were positively correlated with the majority of immune cells according to the correlation heatmap. However, the correlations of YTHDF3, WTAP, and HNRNPC with immune cells were significantly weaker than that of LRPPRC (Additional file 1, Figure S2). Taken together, LRPPRC is likely to be the core molecule that determines the m6A cluster of MI, and the expression level of this gene significantly affects immune cell infiltration. Based on existing studies, however, we were unable to determine whether LRPPRC directly regulates the level of immune cells.

### 3.5. The m6A cluster influences cardiac fibrosis in MI

**Figure 6:**
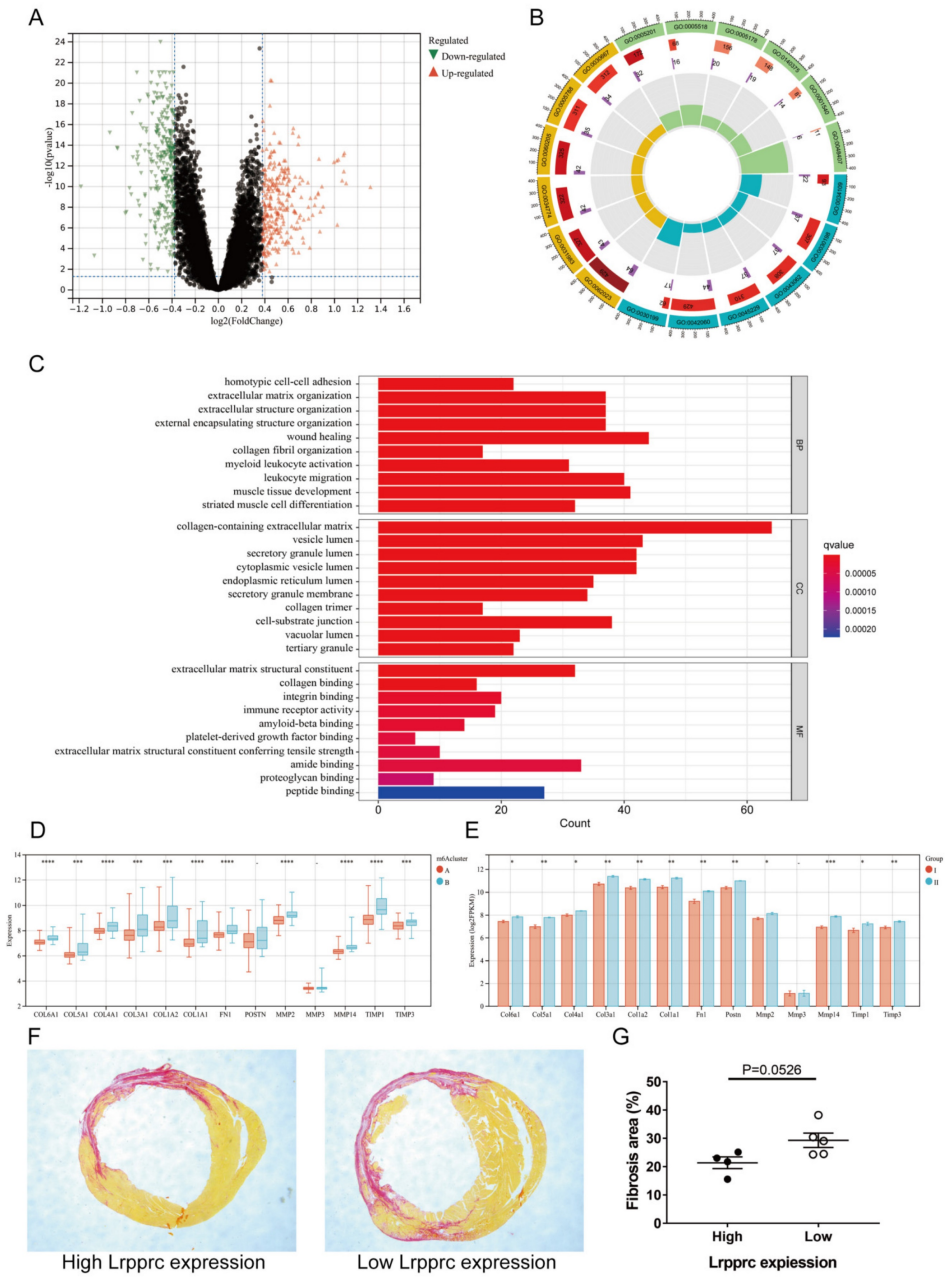
** m6A clusters and cardiac fibrosis in MI.** Volcano plot of the distribution of DEGs between two m6A clusters. (B) GO circle diagram of the DEGs. (C) GO barplot of the DEGs. Different colors represent different *p* values. (BP: biological process; CC: cellular component; MF: molecular function) (D-E) Differences in expression of fibrosis-related genes between the two m6A clusters in human and mouse samples respectively. (The significance of difference is marked as follows: *p < 0.05; **p < 0.01; ***p < 0.001, ****p < 0.0001) (F) Sirius red staining of cardiac sections from mice at 4 weeks after MI. Areas of fibrosis were stained red. (G) Comparison of fibrosis area between Lrpprc high expression group and low expression group.

To further explore the characteristics of m6A subtypes in MI, we extracted 591 DEGs between Clusters A and B (fold change>1.3, p value<0.05, Figure 6A, Additional file 7), and we then performed Gene ontology (GO) enrichment analysis based on these DEGs (Figure 6B-C, Additional file 8). Most of the DEGs were related to wound healing, collagen synthesis and bonding, and extracellular matrix regulation, which are closely related to the occurrence and development of MI. In the early stage of ischemic myocardial infarction, cardiac fibroblasts secrete a large amount of collagen fibers to replace the necrotic myocardium in the infarct area and prevent the heart from rupturing, which is a protective process. However, in the later stage, overactive fibroblasts synthesize excessive collagen fibers, leading to detrimental ventricular remodeling. The immoderate fibrosis of hearts affects the contractility of viable myocardium, decreases cardiac output, and even causes lethal arrhythmias, which may reduce the survival rate of patients with MI^25^. To further demonstrate that these physiological processes are related to m6A regulation, we selected several genes closely related to the synthesis or regulation of collagen and compared the differences in these genes between Clusters A and B of clinical samples and Groups I and II of mouse samples (Figure 6D-E). The results showed that most of these genes were significantly upregulated in Cluster B of clinical samples and Group II of mouse samples. We then established MI models in an additional 10 mice (one died 5 days after surgery) and sacrificed them 4 weeks later to evaluate cardiac fibrosis via Sirius red staining. We also extracted RNA from the heart tissue of these mice and measured Lrpprc expression in each sample by qPCR. As mentioned above, Lrpprc expression was predominant in the grouping of mouse samples. Subsequently, we divided the nine samples into two groups according to the expression level of Lrpprc (4 samples in the high expression group and 5 samples in the low expression group) and then analyzed the difference in fibrosis area between these two groups. The degree of fibrosis in the low expression group was slightly higher than that in the high expression group, which corresponded to the difference in gene expression (Figure 6F-G) (Additional file 1, Table S2, Figure S3).

### 3.6. WGCNA based on DEGs between m6A clusters

**Figure 7:**
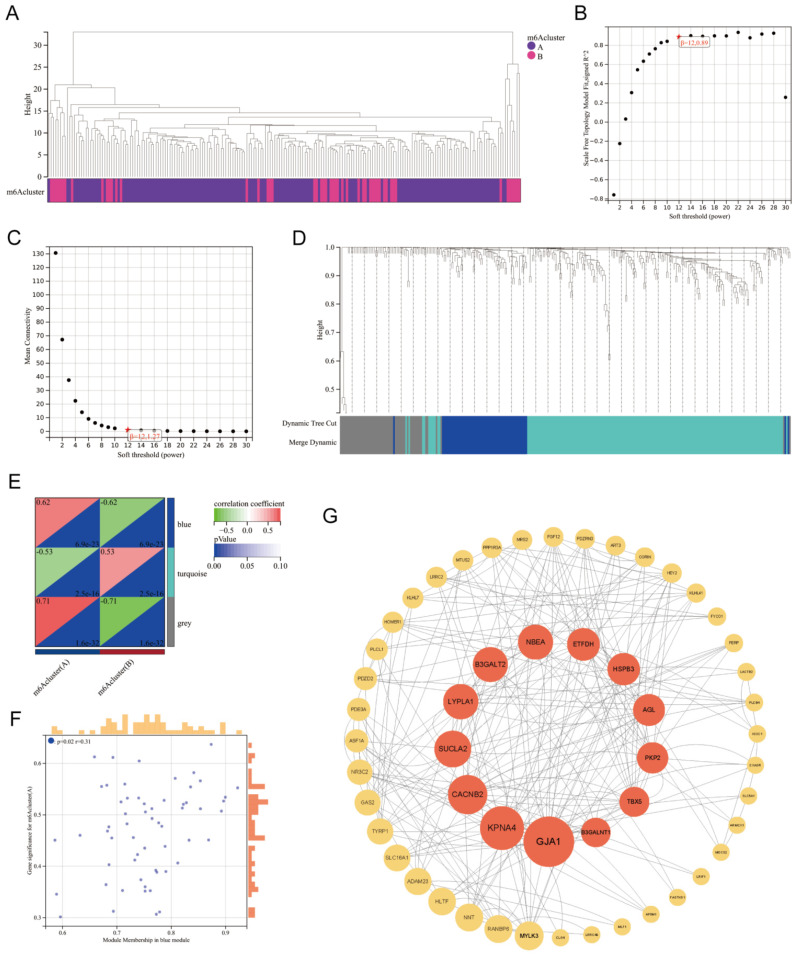
** WGCNA of the DEGs between m6A clusters.** Clustering dendrogram of two m6A subtypes in MI. (B-C) Analysis of the scale-free fitting index and the mean connectivity respectively for soft-thresholding power from 1 to 30. (D) Gene dendrogram obtained by average linkage hierarchical clustering. The color row underneath the dendrogram shows the module assignment determined by the dynamic tree cut, in which 3 modules were identified. (E) Correlation heatmap between module eigen genes and the m6A modification patterns. (F) A scatterplot of gene significance (GS) for m6A modification pattern A vs module membership (MM) in the blue module. (G) A protein-protein interaction (PPI) network based on the genes in the blue module. These genes were arranged according to betweenness centrality and the top 13 genes were marked red.

Based on the DEGs between m6A Clusters A and B, we performed weighted gene coexpression network analysis (WGCNA) (Figure 7A). WGCNA, a systematic biological approach to describe patterns of gene association among different samples, can be used to identify gene sets with highly synergistic variation and to identify candidate biomarker genes or therapeutic targets^26^. According to Figure 7B-C, the lowest soft threshold for constructing a scale-free coexpression network was 12. Subsequently, we established a coexpression network based on the lowest soft threshold and divided the DEGs into the following three different network modules: gray module, blue module, and turquoise module (Figure 7D). Among the modules, the gray module represented the invalid module, which contained all the genes that did not belong to the other two modules. We next analyzed the correlation between the two modules and the m6A cluster (Figure 7E), which demonstrated that the correlation coefficient between the blue module and Cluster A was the highest (r=0.62). To further explore the correlation between the blue module and Cluster A, we obtained gene significance (GS) by calculating their correlation and module membership (MM) by analyzing the correlation between module feature vectors and gene expression (Figure 7F). There was a positive correlation between MM and GS, which indicated that the genes in the blue module were strongly correlated with m6A regulation. We next screened the hub genes in the blue module (|MM|>0.4 and |GS|>0.1) and constructed a protein‒protein interaction (PPI) network using Cytoscape to observe the interaction between these genes (Figure 7G). These genes were arranged according to betweenness centrality, and the gene with the highest betweenness centrality was GJA1, which suggested that this gene may be a core protein in the PPI network. GJA1, also known as CX37, encodes a protein that is a component of gap junctions, an array of intercellular channels that provide a pathway for the diffusion of low molecular weight substances between cells. Mutations in this gene are associated with atherosclerosis and a higher risk of MI^27^, ^28^. Undoubtedly, the link between MI and GJA1 cannot be ignored, and GJA1 is likely to play an important role in the m6A regulation of MI.

## 4. Discussion

In the present study, we systematically analyzed the changes and regulatory patterns of m6A regulators in heart tissues of MI by combining bioinformatics analysis with animal experiments. We identified 12 differentially expressed m6A genes (WTAP, ZC3H13, RBM15B, YTHDC1, YTHDF1, YTHDF3, HNRNPC, FMR1, LRPPRC, IGFBP1, ELAVL1, and FTO) between MI patients and healthy controls and used them to establish a disease diagnosis model of MI using the random forest method, and we validated the feasibility of this model. Nearly all types of immune cells are involved in inflammation and ventricular remodeling after MI, and their quantity changes dynamically throughout the process of the disease^29^. It is generally believed that T cells, monocytes, macrophages, and dendritic cells play a relatively more important role in hearts with chronic ischemic heart failure, and their functions in maintaining the chronic inflammatory response in the cardiac microenvironment have not been fully explored^30^. Therefore, we analyzed the correlation between these m6A genes and the infiltration of immune cells in the MI samples and found that LRPPRC was negatively correlated with most immune cells, among which MDSCs and Tregs showed the greatest difference between the high and low LRPPRC expression groups. We then used an unsupervised clustering method to classify MI samples into m6A clusters, namely, Clusters A and B. Compared to Cluster A, LRPPRC, YTHDF3, and FMR1 expression was significantly reduced in Cluster B. Through the analysis of the difference in immune cell infiltration between these two clusters, we found that most immune cells had a higher degree of infiltration in Cluster B and that the intercluster difference in MDSCs and Tregs was more pronounced. Interestingly, similar results were verified in mice. Lrpprc was highly differentiated in the two groups of mouse samples (Groups I and II), which were subsequently analyzed for immune cell infiltration. As expected, Group II, characterized by low Lrpprc expression, revealed higher infiltration of immune cells, especially MDSCs and Tregs.

We hypothesized that decreased LRPPRC expression may increase the immune response in the MI heart and ultimately enhance the infiltration of immunosuppressive cells, such as MDSCs and Tregs. LRPPRC is a m6A reader that encodes a leucine-rich protein with multiple pentapeptides. The exact function of this protein is still unclear, but it has been reported to play a role in cytoskeletal organization, vesicle transport, or transcriptional regulation of nuclear and mitochondrial genes^31^. LRPPRC is required for the coordination of polyadenylation and translation of mitochondrial mRNA^32^. At present, studies have shown that this gene is associated with a variety of diseases, including atherosclerosis, Parkinson's disease, and tumors^33^, ^34^. However, there have been no studies on the relationship between LRPPRC and immune cells so far. Both MDSCs and Tregs are important immunosuppressive cells and are closely related to the occurrence and development of cardiovascular diseases. MDSCs are thought to be produced in chronic inflammation, especially in advanced cancer, and possess T cell immunosuppression capabilities^35^. The use of MDSC inhibitors in tumor patients enhances T cell immunity and improves antitumor properties^36^. MDSCs also inhibit cardiomyocyte hypertrophy and maintain cardiac function in patients with heart failure^37^. Tregs function in almost all cardiovascular diseases. Researchers have found that increased Treg recruitment in the hearts of mice with MI effectively alleviates inflammation, reduces the infarcted area, and improves the survival rate^38^, ^39^. Tregs also inhibit cardiac fibroblast activation after acute MI, thereby reducing cardiac fibrosis^40^. However, it remains unknown whether LRPPRC is directly related to the increase in MDSC and Treg infiltration, and additional studies are needed to why LRPPRC reduces immune cell infiltration in MI hearts.

We also screened DEGs between Clusters A and B, and we performed GO enrichment analysis and WGCNA on the basis of these genes. GO enrichment analysis indicated that many of the DEGs were enriched in biological processes related to collagen synthesis and wound healing, which was verified in clinical and mouse samples. These results indicated that m6A regulators may be involved in cardiac fibrosis. Considering that the cardiac fibrosis level is usually positively correlated with the inflammation level in MI, we speculated that the higher expression of fibrosis genes in Cluster B may be attributed to its higher proinflammatory immune cell infiltration. In the following WGCNA, we screened the most significant gene expression modules in the two clusters and mapped the protein‒protein interaction network, which indicated that GJA1 might be the central point of the PPI network. Because the relationship between GJA1 and the development of atherosclerosis has been previously reported, these findings suggested that GJA1 could be an important molecule mediating m6A regulation and the pathogenesis of MI.

In summary, the present study indicated that LRPPRC-dominated m6A regulation significantly affects the immune response and fibrosis degree in MI heart tissue. However, the present study had several limitations. We only obtained the total gene expression data in heart tissues from the GEO database and did not obtain the specific clinical information of patients. Clinical indicators closely related to MI, such as cardiac function (cardiac output and ejection fraction), BNP level, and coronary angiography, were not available. In addition, the infiltration level of each immune cell type in heart tissues was only evaluated by ssGSEA, which cannot precisely reflect the cardiac immune response state. Therefore, we were unable to conclude that each element of this study was correlated with each other. In addition, the number of mice used to verify the clinical sample analysis results was small, leading to low significance of some results. Although most of the verification results were consistent with the clinical samples, we still lacked sufficient animal experiments for verification. Further, pure bioinformatics analysis was unable to explore the specific regulatory mechanism of m6A underlying inflammation and fibrosis. Therefore, additional studies of knockout mice are warranted to understand how m6A regulation affects the occurrence and development of MI.

## Supplementary Material

Supplementary: Additional file 1 - supplementary tables and figures; Additional file 2 - the complete gene expression matrix of the clinical samples; Additional file 3 - m6A clusters of the clinical MI samples; Additional file 4 - gene expression matrix of the mouse hearts with MI model; Additional file 5 - list of gene sets of 23 immunocytes; Additional file 6 - ssgsea score of 23 immunocytes in the clinical samples; Additional file 7 - list of DEGs between cluster A and B; Additional file 8 - the enriched GO terms of DEGs between cluster A and B.

## Abbreviations

m6A: N6-methyladenosine
MI: myocardial infarction
RF: random forest
SVM: support vector machine
Treg: regulatory T cell
MDSC: Myeloid-derived suppressor cells
ssGSEA: single-sample gene-set enrichment analysis
PCA: principal component analysis
DEGs: differentially expressed genes
GO: Gene Ontology
WGCNA: weighted gene co-expression network analysis
MM: module membership
GS: gene significance
