# Supplementary material for: Integrative Analysis of N6-methyladenosine RNA modifications related genes and their Influences on Immunoreaction or fibrosis in myocardial infarction
Source: Int J Med Sci. 2024 Jan 1;21(2):219–33. doi: 10.7150/ijms.86210 (PMC10758152; doi:10.7150/ijms.86210)
Supplement: Supplementary file 1 — Supplementary: Additional file 1 - supplementary tables and figures; Additional file 2 - the complete gene expression matrix of the clinical samples; Additional file 3 - m6A clusters of the clinical MI samples; Additional file 4 - gene expression matrix of the mouse hearts with MI model; Additional file 5 - list of gene sets of 23 immunocytes; Additional file 6 - ssgsea score of 23 immunocytes in the clinical samples; Additional file 7 - list of DEGs between cluster A and B; Additional file 8 - the enriched GO terms of DEGs between cluster A and B. [file ijmsv21p0219s1.zip › Additional file 1, supplementary tables and figures.docx]

**Table A1.** Classification of m6A regulators.

| **Categories of m6A regulators** | **Gene name** |
| --- | --- |
| Methyltransferases (writers) | METTL3, METTL14, METTL16, WTAP, VIRMA, ZC3H13, RBM15, RBM15B, CBLL1 |
| Demethylases (erasers) | FTO, ALKBH5 |
| Methylation readers (readers) | YTHDC1, YTHDC2, YTHDF1, YTHDF2, YTHDF3, HNRNPC, FMR1, LRPPRC, HNRNPA2B1, IGFBP1, IGFBP2, IGFBP3, RBMX, ELAVL1, IGF2BP1 |

**Table A2.** Grouping of the mouse samples for Sirius red staining.

| Group | Lrpprc expression | Fibrosis area (%) |
| --- | --- | --- |
| High Lrpprc expression | 1.546551 | 15.57093426 |
|  | 1.013901 | 25.14220705 |
|  | 0.675906 | 38.23279524 |
|  | 0.990453 | 29.05620361 |
| Low Lrpprc expression | 0.860992 | 24.31749242 |
|  | 1.290388 | 21.73633441 |
|  | 1.035672 | 23.04126101 |
|  | 0.907627 | 30.42357274 |
|  | 0.678510 | 24.46852855 |

**Table A3.** Primer sequences used for qPCR

| Hnrnpc-F | CATTGGGAATCTCAACACTCTGG |
| --- | --- |
| Hnrnpc-R | CAAATGAGGAACCGTACATCTCC |
| Fto-F | TTCATGCTGGATGACCTCAATG |
| Fto-R | GCCAACTGACAGCGTTCTAAG |
| Zc3h13-F | ATCCCGAAGACCTAGCGTATT |
| Zc3h13-R | TGAAGGGCCATGTATGAACCT |
| Fmr1-F | CATGATGTGAGATTCCCACCAC |
| Fmr1-R | GGGATTAACAGATCGTAGACGC |
| Wtap-F | GAACCTCTTCCTAAAAAGGTCCG |
| Wtap-R | TTAACTCATCCCGTGCCATAAC |
| Lrpprc-F | GTAGCCCAGGGAGCAATCAAG |
| Lrpprc-R | GACACCTAACTGCTGAAGTTTGT |
| Ythdf3-F | GATCAGCCTATGCCATATCTGAC |
| Ythdf3-R | CCCCTGGTTGACTAAAAACACC |
| Ythdc1-F | GTCCACATTGCCTGTAAATGAGA |
| Ythdc1-R | GGAAGCACCCAGTGTATAGGA |


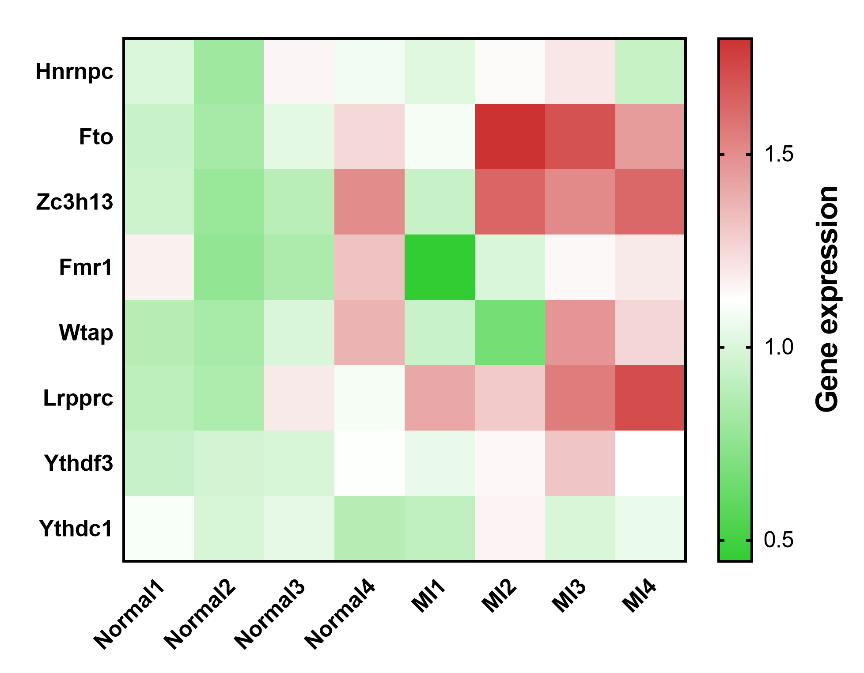


**Figure A1.** Heatmap of the expression of the top eight most important m6A genes in mice.


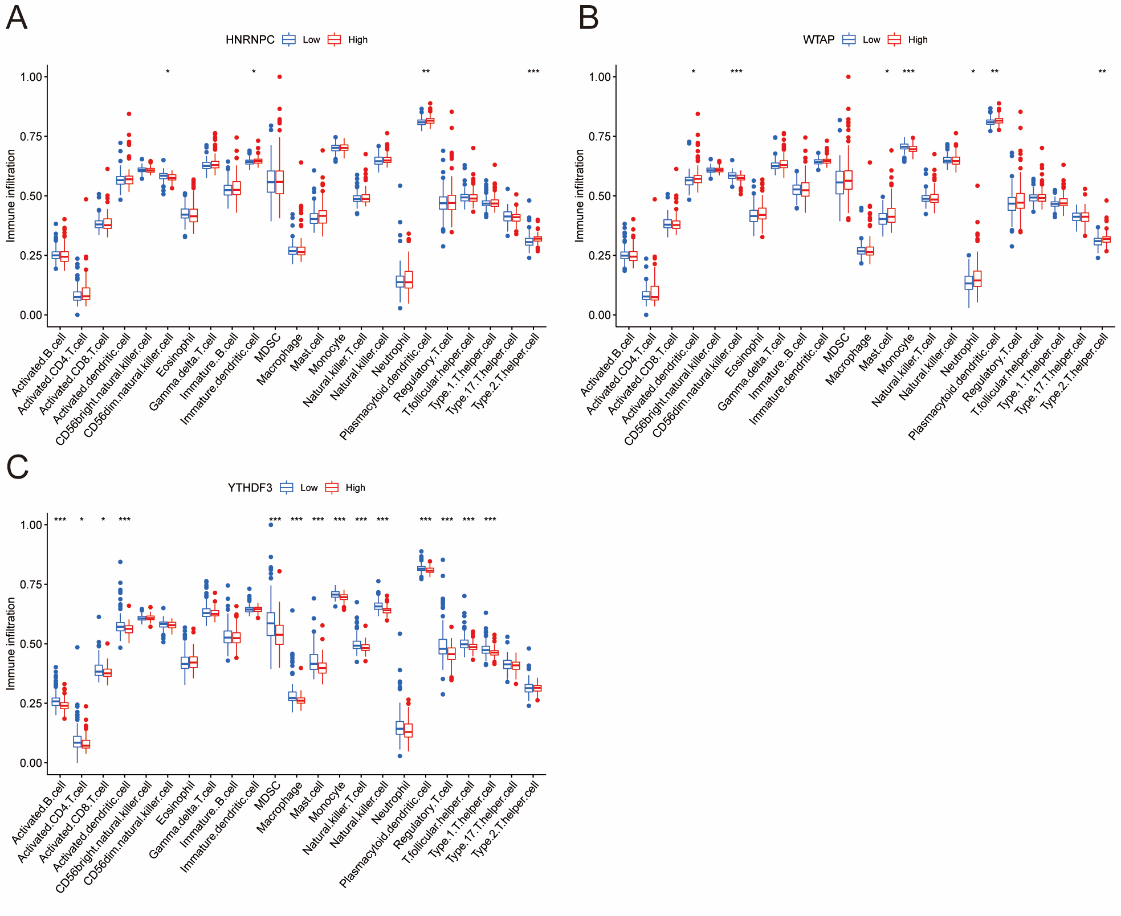


**Figure A2.** Differences in infiltration of immune cells between the high and low expression group of HNRNPC, WTAP and YTHDF3 respectively.

**
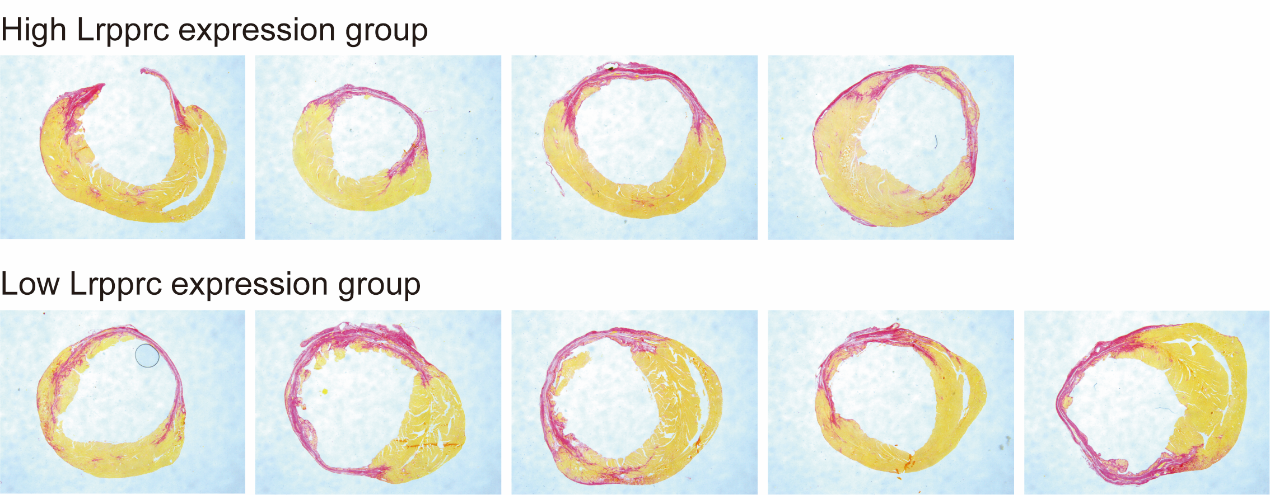
**

**Figure A3.** Sirius red staining of cardiac sections from mice at 4 weeks after MI (All samples).
